# Mr. Squealer the Rat-Catcher

**Published:** 1917-03-17

**Authors:** 


### THE EDITOR'S LETTER-BOX.

### MR. SQUEALER THE RAT-CATCHER.

*To the Editor of* The Hospital.

Sir.—*Truth* is unremitting in its efforts on behalf of Mr. Barker, the bone-setter. As the military authorities harden their hearts and are deaf to the heart-rending appeals of *Truth*, and even go so far as to shelter themselves behind a ridiculous law, which, it appears, prevents the bone-setter from receiving official status in the Army, *Truth* demands an Act of Parliament to compel them to admit him! Well, well! Why only an Act of Parliament? Why not a special meeting of representatives of the oversea Dominions?—Why not a European Congress of representatives of all the Allied Powers? Shall we wait until America declares war, and Mr. Wilson is recovered from his indisposition, or is the matter too urgent?

And while they are about it there is another gross injustice that might be remedied at the same time. It is well known that the trenches are swarming with rats, and many professional rat-catchers have failed lamentably and disgracefully to cope with the plague. We are acquainted with a most skilful amateur rat-catcher, to whom the Pied Piper of Hamelin was but a bungling amateur. We demand that he shall at once be employed in the Army, and receive military status, with a sufficiently noble uniform, with plenty of stripes and stars and crowns, and an abundance of pockets to put the rats in. The trade union of professional rat-catchers will, of course, object. Away with them! Crucify them! Bang them over the head with an Act of Parliament! The rat-catcher we recommend is Our rat-catcher. We have taken him up, and we mean to see this matter through. Lord Derby looks upon him with the sentiments with which a trade unionist looks upon a blackleg. He ought to be above that sort of thing, especially when he sees what many of the rat-catching profession are. The truth of the matter is that there is no obstacle of the slightest substance except the prejudice of the rat-catching profession against recognising any skill or knowledge which is not branded with an academic hall-mark. This is the question which the House of Commons and the country and the Allied Governments have to face, and which we trust they will face. Mr. Squealer the rat-catcher is a grossly ill-used person, and we mean to see him righted.—I am Sir, in mercy, A Friend of the House of Commons.

